# Novel substituted methylenedioxy lignan suppresses proliferation of cancer cells by inhibiting telomerase and activation of c-*myc* and caspases leading to apoptosis

**DOI:** 10.1038/sj.bjc.6600422

**Published:** 2002-07-15

**Authors:** P Giridharan, S T Somasundaram, K Perumal, R A Vishwakarma, N P Karthikeyan, R Velmurugan, A Balakrishnan

**Affiliations:** Centre for Biotechnology, Anna University, Chennai 600 025, India; Bio-organic Chemistry Lab, National Institute of Immunology, New Delhi 110 067, India; Maruthi Biotech, Chennai 600 034, India

**Keywords:** *in vitro* bioscreen, *P urinaria*, telomerase, tubulin, apoptosis

## Abstract

Conventional solvent fractionation and bioactivity based target assays were used to identify a new anti-cancer molecule from *Phyllanthus urinaria*, a herbal medicinal plant used in South India. At each step of the purification process the different fractions that were isolated were tested for specific anti-proliferative activity by assays measuring the inhibition of [^3^H]thymidine incorporation, and trypan blue drug exclusion. The ethyl acetate fraction that contained the bioactivity was further purified and resolved by HPLC on a preparative column. The purity of each of the fractions and their bioactivity were checked. Fraction 3 demonstrated a single spot on TLC and showed maximum anti-proliferative activity. This fraction was further purified and the structure was defined as 7′-hydroxy-3′,4′,5,9,9′-pentamethoxy-3,4-methylene dioxy lignan using NMR and mass spectrometry analysis. The pure compound and the crude ethyl acetate fraction which showed anti-proliferative activities were examined for ability to target specific markers of apoptosis like *bcl2,* c-*myc* and *caspases* and for effects on telomerase. Four specific cancer cell lines HEp2, EL-1 monocytes, HeLa and MCP7 were used in this study. The results indicate that 7′-hydroxy-3′,4′,5,9,9′-pentamethoxy-3,4-methylene dioxy lignan was capable of inhibiting telomerase activity and also could inhibit *bcl2* and activate caspase 3 and caspase 8 whose significance in the induction of apoptosis is well known. We believe that this compound could serve as a valuable chemotherapeutic drug after further evaluations.

*British Journal of Cancer* (2002) **87**, 98–105. doi:10.1038/sj.bjc.6600422 www.bjcancer.com

© 2002 Cancer Research UK

## 

Traditional herbs have been used as a valuable source of medicines for the treatment of several diseases in many parts of the world. In this study, we have examined the potential anti-cancer property of *Phyllanthus urinaria*, which has been used for treatment of jaundice, asthma and cancer (Mulchandani and Hassarajani, 1984).

The role of apoptosis in cancers has been studied in detail, apoptosis being a highly regulated cellular response with crucial checkpoints regulating the fate of cells. These checkpoints are processing centers sensing extracellular signals, amplifying localised signals, integrating information from these cells and directing them towards death cascade (Thompson, 1995). Several proto-oncogenes such as c*-myc*, *bcl2,* c-*fos* (Evan *et al*, 1992) have been implicated as active apoptotic effectors, while cH-*ras* and v-*src* are known to modulate non-direct regulating interactions. Using these effectors as targets for new drug development, several new compounds with different chemical entities have been identified from medicinal plants. A wide variety of natural substances have been recognised to induce apoptosis in various tumour cells of human origin (e.g., Liu *et al*, 2000).

The role of c-*myc* in apoptosis is highlighted in many human cancers (Evan and Littlewood, 1998). The observation that c-*myc* actively promotes apoptosis explains the potent cooperative effects observed between c-*myc* and *bcl2* (Strasser *et al*, 1990). One of the targets of oncogene -induced sensitisation is the mitochondria and c-*myc* facilitates cytochrome *c* release from the mitochondria (Juin *et al*, 1999). The released cytochrome *c* activates caspases, a family of cysteine proteases and *bcl2* suppression thereby causing apoptosis. Senescence is an irreversible programme of cell cycle arrest that is disturbed in many tumours or tumour derived cell lines (Barnett *et al*, 1993). Owing to the end replication problem, telomeres shorten during each cell division unless telomerase is expressed. In cancers telomerase is found and is known to play a role in the cancer cell to evade apoptosis (Kim *et al*, 1994; Broccoli *et al*, 1995). The use of telomerase as a possible target for anticancer drug development seems to be promising. The interplay between telomerase and key players of the apoptotic cascade will provide evidence of the possible mode of induction of cell death using specific chemical entities.

In this paper, we have employed bioactivity-based assays to identify anti-proliferative potential of the extracts of the medicinal plant *P. urinaria* using simple *in vitro* methods. Bioassay-guided fractionation has enabled us to obtain a pure compound with anti-cancer activity. Its molecular structure was elucidated as 7′-hydroxy-3′,4′,5,9,9′-pentamethoxy-3,4-methylenedioxylignan.

## MATERIALS AND METHODS

### Chemicals and reagents

All cell lines used in this study were obtained from ATCC. All fine chemicals were obtained from Sigma- Aldrich, St Louis, MO, USA and USB, Cleveland, OH, USA. [^3^H]thymidine was obtained from Amersham, UK. MTS assay kit was procured from Promega, USA. TRAP assay and Teloquant Kit were obtained from Pharmigen, USA. Bcl2 antibody were obtained from Santa Cruz Biotechnology, Inc. Santa Cruz, California and caspases 3 and 8 antibodies were obtained from BD PharMingen, USA. *Phyllanthus urinaria* was obtained from South India. The species was examined by a taxonomist to confirm the same. Different batches were obtained, processed and checked for similar profile of the extracts by TLC.

### Solvent extraction

The dried plant powder (100 gram) of *P.urinaria* was extracted with different solvents at room temperature, from non-polar to polar solvents namely ethylene glycol, ethyl acetate, methanol and water. Each of these extracts were concentrated in a rotatory evaporator under reduced pressure, giving 2–3 gram of each individual extracts. Ten mg of the dried powder from each of the solvent extracts were reconstituted to 1 ml with the respective solvents and they were serially diluted to 1 : 10, 1 : 50, and 1 : 100 of the original stock preparations for anti-proliferative studies.

### Cell culture

HEp-2 (alveolar epithelial carcinoma cell line), MCF7 (Breast cancer cell line), HeLa (Cervical cancer line) and EL-1 monocyte cells were maintained in F-12 Dulbecco's Modified Eagle Medium (DMEM) supplemented with 10% serum amphotericin (3 μg ml^−1^), gentamycin (400 μg ml^−1^), streptomycin (250 μg ml^−1^), penicillin (250 units ml^−1^) in a carbon dioxide incubator at 5% CO_2_.

### [^3^H]thymidine incorporation studies

[^3^H]thymidine (1 μCi per 1 ml of medium) was added to the medium in which the cell line was maintained, a day prior to the addition of the extracts. The different solvent fractions were added to the cells. In a six well plate 20 μl (10 mg ml^−1^) of sample was added to all wells that contain 1 ml of medium. As controls the same volume of the different solvents was added. Different dilutions of 1 : 10, 1 : 50 and 1 : 100 of the ethyl acetate fraction was also carried out. The cultures were trypsinised at the desired time points, pelleted and washed sequentially with 10% and 5% TCA and solubilised in 0.1 N NaOH and 0.025% SDS solution. The radioactivity of the samples was measured in the WALLAC 1409 Liquid scintillation counter and expressed as CPM mg^−1^ protein.

### Thin layer chromatography (TLC)

TLC analysis was done with each of the solvent extracts. Four types of solvent systems were used: (a) 25% ethyl acetate in hexane, (b) 50% ethyl acetate in hexane, (c) 100% ethyl acetate and (d) 5% methanol in ethyl acetate.

### Northern analysis

HEp-2 cells were grown in six well plates for 24 h; mRNA was extracted from the cells using 1 ml TriZol reagent followed by chloroform-isopropanol extraction. Approximately 50 μg of the RNA was denatured by heating at 65°C for 10 min and loaded on to a 1.2% formaldehyde-agarose gel and run at 100 V for 1 h. RNA was transferred to a nitrocellulose paper by upward capillary transfer, UV cross-linked and stored at 4°C until further probing. Plasmids bearing DNA probes for the proto-oncogene c-*myc was* gifted by Prof Peter Williams, Leicester University, UK. DNA probes were used at 150 μg ml^−1^ of hybridisation buffer and labelled with [^32^P]dCTP using Rediprime kit (Amersham Life Sciences).

### Column chromatography

Column was packed with hexane using silica gel 100–200 mesh size as a matrix, samples were loaded as dried slurry of silicagel and the column was eluted with increasing concentration of ethyl acetate and methanol to increase polarities. The ratio of material loaded and silica gel was 1 : 20. The fractions were analysed by pre-coated TLC plates (Merck) and spots were visualised by exposure to iodine, UV and phosphomolybdic acid spray reagents. The active fraction was further purified by reverse phase HPLC using acetonitrile-water gradient system; the compounds were detected by UV-detector at 260 nm. The purity of active compound was established by TLC and HPLC.

### Structural characterisation

**Figure 8:**
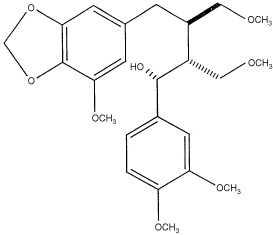
Structure of the pure compound 7′-hydroxy-3′,4′,5,9,9′-pentamethoxy-3,4-methylene dioxy lignan as elucidated by NMR and mass spectrometry analysis.

The structure of the active compound was determined by ^1^H, ^13^C and ^1^H - ^13^C COSY NMR spectroscopy and electrospray ionisation mass spectrometry. The NMR experiments were done in CDCl_3_ solution and ESMS using acetonitrile-water spray condition. The ^1^H and ^13^C NMR data showed characteristic peaks for lignan skeletons and confirmed presence of five OMe groups (δ 3.7–3.8 p.p.m., 5×OCH3). The presence of methylenedioxy was due to a signal at 5.10 p.p.m. The positioning of OMe groups in ring B (lower ring) was ascertained by the coupling pattern in ^1^H NMR data and comparisons in the repeated data of related lignans. The mass spectra confirmed elemental composition C_24_H_32_O_8_ (448). Full details of structural characterisation will be published elsewhere (Figure 8

).

### Non isotopic telomeric repeat amplification protocol (TRAP) assay

Lysate preparation and the TRAP assay were performed as described earlier (Kim and Wu, 1997). Briefly, HEp-2 and EL1-monocytes are lysed in 200 μl of ice-cold CHAPS lysis buffer. After 30 min of incubation on ice, the lysates were centrifuged at 14 000 *g* for 60 min at 4°C and the total protein concentration of the supernatant standardised according to the Bradford method (approximately 1 μg 1 μl^−1^). Six microlitres of supernatant were used for the TRAP assays. After an initial incubation period (30°C for 30 min), telomerase products were amplified using TS and RP primers (35 cycles at 94°C for 30 s, 53°C for 30 s and 72°C for 30 s). Assay specificity was confirmed by inclusion of RNAase pre-incubation control step, and Taq inhibition was checked by including the 36 bp internal control. Amplicons were electrophoresed on a 12.5% non-denaturing polyacrylamide gel (19 : 1) stained with ethidium bromide and analysed.

### Tubulin assay

HEp-2 cells were exposed to both crude ethyl acetate fraction (10 μg ml^−1^) and pure compound (4.46 μM) for 24 h. The treated monolayers were fixed with 3% paraformaldehyde in PBS for 30 min. The fixed cells were then permeabilised with 0.1% Triton X-100 in PBS at 4°C for 5 min. The cells were washed three times with PBS and treated with anti-tubulin antibody (dilution in PBS 1 : 200) and incubated at 37°C for 30 min. The cells were then blocked with 5% serum to avoid non-specific binding. The cells after blocking were washed in PBS twice. The cells were then treated with secondary antibody to tubulin, FITC labelled antimouse IgG for 30 min at 37°C.

### Immunoblot analysis

Total cell lysates were prepared as reported in our earlier work (Narayanan *et al*, 2000). Proteins were separated on SDS 10% polyacrylamide gel. The gel was transferred onto a nitrocellulose membrane (Hybond C+, Amersham Life Sciences) at 220 mA for 3 h. The membrane was then washed three times with PBS and blocking reagent (3% Skimmed Milk) was added and blocked overnight at 4°C. Blocking reagent was washed with PBS twice for 5 min each and primary antibody was added (bcl2, caspase 3 and 8). One per cent BSA in PBS, 0.1% Tween 20 was added to the blot along with the primary antibody (1 : 2000) and rocked gently at room temperature for 1 h. The blot was washed three times with PBS for 5 min each. Secondary antibody (1 : 5000) in 1% BSA in PBS, 0.1% Tween 20 was allowed to hybridise for 1 h at room temperature. The bands were detected using the chromogenic substrate NBT-BCIP in alkaline phosphate buffer.

### Measurement of apoptosis by flow cytometry

For flow cytometric analysis 1.5×10^6^ cells were processed as follows. Cells were lysed with 0.1% Triton X-100 and incubated with 50 μg ml^−1^ RNase A for 30 min at 37°C. Nuclear DNA was stained with 50 μg ml^−1^ propidium iodide (PI) and cells were analysed for DNA content in FACScan flow cytometer equipped with cell quest software (Becton Dickinson USA).

## RESULTS

### Extraction and column purification

**Figure 1:**
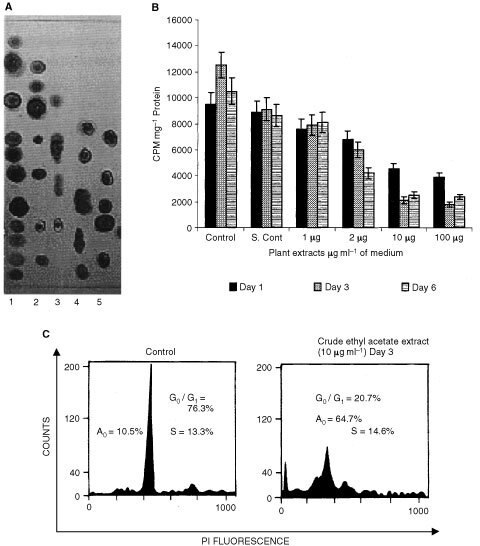
(**A**) Thin layer chromatography profile of each of the solvent extracts. (1) Ethyl acetate extract (2) Ethylene glycol extract, (3) Acetone extract, (4) Methanol extract, (5) Water extract. Products are resolved in 50% ethyl acetate and 50% methanol and visualised in UV at 260 nm and Iodine chamber. (**B**) Anti-proliferative effect of the ethyl acetate extract on HEp-2 cells monitored by [^3^H]thymidine incorporation at 1, 3 days and 6 days time points. Different dilutions of the same fractions were tested. Control is untreated cells. S. Cont, is solvent control. Values are mean±s.e. (means of three replicates). (**C**) Flow cytometry analysis of HEp2 in control and treated cells with crude ethyl acetate extract (10 μg ml^−1^) at 72 h.

The various solvent extracts were collected and each fraction was tested for bioactivity. The TLC analysis of all the different fractions were run under conditions described. Figure 1A

shows the TLC analysis of each solvent extract.

### Anti-proliferative studies

The different solvent extracts of *P. urinaria* were added to the cells as described above. Thymidine incorporation studies between two time points, day 1 and day 3, showed marked inhibition of proliferation of HEp2 cells by the ethyl acetate extract. Hence we followed the ethyl acetate extract for the various studies. When the dilutions were increased the extent of inhibition of proliferation was reduced in ethyl acetate extract. The maximum inhibition of proliferation was seen in crude ethyl acetate extract at 100 μg ml^−1^ and in 10 μg ml^−1^ of medium (Figure 1B). The extracts of the other solvents namely ethylene glycol, acetone, methanol and water extracts were also tested, since the inhibition of proliferation was maximum with ethyl acetate fractions, this was taken up for further purification.

### Purification of active fraction

**Figure 2:**
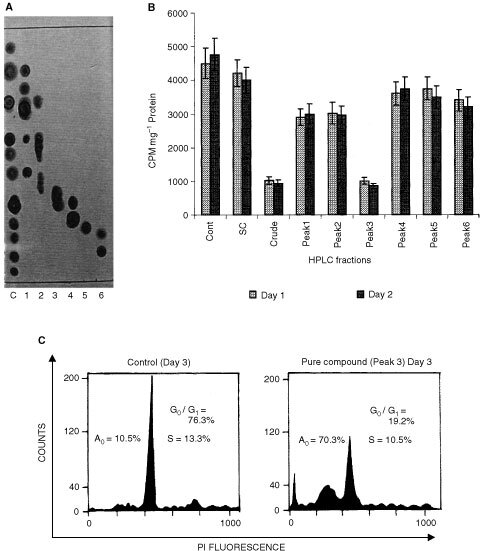
(**A**) Purification of ethyl acetate fraction by HPLC, six peaks were obtained and they were analysed by TLC and visualised under UV at 260 nm. (**B**) Growth inhibition studies of the six peaks on HEp2 cells by [^3^H]thymidine incorporation . The cells were treated with 2 μg ml^−1^ of all peaks. Values are mean±s.e. (means of three replicates). (**C**) Flow cytometry analysis of the HEp2 in control and treated cells with pure compound 4.46 μM (Peak 3) at 72 h.

**Figure 3:**
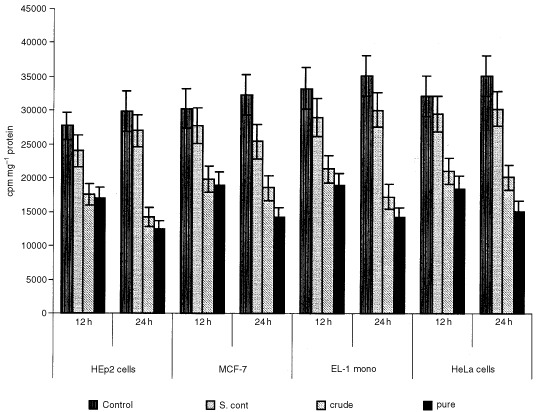
Anti-proliferative effect of the crude ethyl acetate fraction (10 μg ml^−1^) and pure compound (4.46 μM) on HEp2, MCF-7, EL1 monocytes and HeLa cells at day 1 and day 2.

Various concentrations of hydrocarbons was tested on the TLC, for the best resolution. Hexane/ethyl acetate (3 : 1) gave good separation. The above mentioned solvent system was used to elute the active compound. The fraction was concentrated under reduced pressure and the homogeneity of the compounds were determined by TLC. In the C-18 HPLC column using similar conditions as above, six fractions were obtained and purity was checked by TLC (Figure 2A

), Compound 3 showed maximum activity on HEp2 cells in 24 h (Figure 2B). This fraction was subjected to structural characterisation studies and the molecular weight and structure of the lead compound was determined using ESMS and NMR studies. The IC_50_ value of pure compound in Hep2 cells was found to be 4.46 μM by establishing a concentration-dependence curve (data not shown). Therefore in all the experiments performed with the pure compound, we used the dose of 4.46 μM. The crude ethyl acetate extract (10 μg ml^−1^) and pure compound (4.46 μM) also showed marked inhibition on HEp2, HeLa, MCF-7 and EL1 monocyte cell lines (Figure 3

). Both the purified compound and crude ethyl acetate extract though cytotoxic to the above four cancer cell lines. However, initial studies on normal human lymphocytes, did not show cytotoxic effects at the same doses which were used on cancer cell lines. A more detailed analysis is currently being carried out using primary culture from normal biopsy samples from different tissues.

### Northern analysis

**Figure 4:**
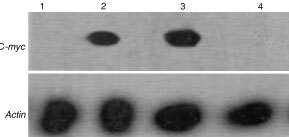
Northern blot analysis of c-*myc* at 24 h. The cells were exposed to the crude ethyl acetate extract (10 μg ml^−1^) (Lane 2); Pure compound (4.46 μM) (Lane 3); The untreated cells as control (Lane 1) and solvent control (Lane 4) showed no induction of c-*myc* mRNA. Actin was used as house keeping.

HEp-2 cells were treated with crude ethyl acetate fraction (10 μg ml^−1^) and the purified compound (4.46 μM) for 24 h. c-*myc* expression levels in the above two conditions were elevated after 24 h of treatment (Figure 4

).

### Assessment of telomerase activity through TRAP assay

**Figure 5:**
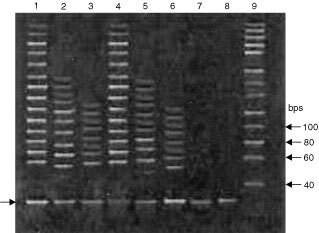
Assessment of telomerase activity by TRAP assay at 12 h.−HEp-2 Untreated control (Lane 1), Treated with crude ethyl acetate extract (10 μg ml^−1^) (Lane 2), Treated with pure compound (4.46 μM) (Lane 3), Control untreated EL-1 monocytes (Lane 4). Treated with crude ethyl acetate extract (10 μg ml^−1^) (Lane 5). Treated with pure compound (4.46 μM) (Lane 6). RNAse treated (-ve)control (Lane 7). Pre incubated at 95° C (Lane 8) and Marker (Lane 9).

Telomerase activity was monitored in HEp-2 and EL1 monocytes cell lines. On treatment with both crude ethyl acetate extract (10 μg ml^−1^) and pure compound (4.46 μM), inhibition of telomerase activity after 12 h of treatment was noted. In case of pure compound, telomerase inhibition was higher than crude ethyl acetate extract (Figure 5

). In the TRAP assay, activity of the telomerase enzyme was assessed with the help of a number of TRAP products formed and also depends on the intensity of the band. The purified compound caused a significant decrease in the activity of telomerase.

### Tubulin assembly assay

**Figure 6:**
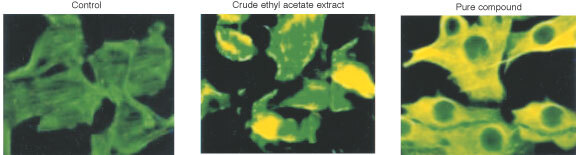
The immunohistochemistry of microtubule assembly shows mitotic arrest in HEp2 cells treated with crude ethyl acetate extract (10 μg ml^−1^) and pure compound (4.46 μM) at 24 h.

7′-hydroxy-3′,4′,5,9,9′-pentamethoxy-3,4-methylene dioxy lignan (4.46 μM) and crude ethyl acetate fraction (10 μg ml^−1^) promote assembly of microtubules after 24 h in HEp2 cells. In immunohistochemistry studies we found mitotic arrest, which are stained in yellow shade (Figure 6

).

### Immunoblot analysis

**Figure 7:**
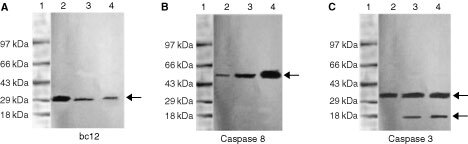
Western blot analysis to confirm the activity of the apoptotic pathway via (**A**) bcl2 inhibition ; (**B**) caspase 8 activation and (**C**) activation of caspase 3 during treatment with the both crude ethyl acetate extract (10 μg ml^−1^) (lane 2) and Pure compound (4.46 μM) (Lane 3), (Lane 1) is an untreated control cells at 36 h.

The bcl2, a family of channel regulators, controls mitochondrial permeability. In our Western blot analysis we found suppression of bcl2 (Figure 7

) expression upon addition of pure compound (4.46 μM) and crude ethyl acetate extract (10 μg ml^−1^) after 36 h as compared to untreated control cells. Subsequently we found elevated level of expression of caspase 8 (Figure 7), which lead to turn on procaspase 3 to active caspase 3 (Figure 7) in treated HEp2 cells. Caspase 3 is an effector caspase that performs the majority of protein cleavages associated with apoptosis.

### FACScan analysis

Apoptosis in the presence of both crude ethyl acetate extract and pure compound has been conformed and quantitated by flow cytometry. A distinct sub-G1 peak is seen in apoptosis, which is due to accumulation of dying cells with reduced amounts of DNA. Our observations show that more than 60% of cells undergo apoptosis after 72 h in HEp2 cells treated with 10 μg of crude ethyl acetate extract per ml of medium (Figure 1C). In case of pure compound (4.46 μM) more than 70% of cells were seen in A_0_ peak (Sub G1) was obtained at 72 h (Figure 2C).

### Structural characterisation

Based on the data generated by the proton, carbon and COSY NMR spectroscopy and electroscopy ionisation mass spectroscopy the structure of the active compound was identified as 7′-hydroxy-3′,4′,5,9,9′-pentamethoxy-3,4-methylene dioxy lignan with a formula indicated in (Figure 8).

## DISCUSSION

Disturbances in signal cascades lead to impairment of cellular functions (Marks *et al*, 2000). Under the influence of a bacteria, virus or mutations as in cancers, improper functioning of several signal molecules has been reported. One of the major defects in cancers is the lack of cells to be driven into the apoptotic mode, due to malfunction of molecules like c-*myc* (Evan and Littlewood, 1998), *ras* (White *et al*, 1995), p53 (Soengas *et al*, 1999), *bcl2* (Haldar *et al*, 1995), *caspases* (Schotte *et al*, 2001) and *telomerase* (Shay and Bacchetti, 1997). Hence, it is thought that targeting such molecules in a cancer may provide a new therapeutic strategy. Traditional medicinal practices of several countries provided valuable leads from which new molecules have been derived and used successfully for the treatment of cancers like etoposide (Karpinich *et al*, 2002). Many of the current chemotherapeutic agents have been obtained from plant sources (Cragg and Newman, 1999).

Our approach has been to exploit the knowledge of traditional medicine practiced in South India to screen plants that have been used for the treatment of cancers. The approach is to integrate chemical extraction and purification of the active molecule to identify the new anti-cancer molecule by determining its bioactivity on known cellular targets that induce apoptosis. By this approach we have been able to isolate and structural elucidate a lignan, 7′-hydroxy-3′,4′,5,9,9′-pentamethoxy-3,4-methylene dioxy lignan from *Phyllanthus urinaria* (Balakrishnan *et al*, 2000).

Thymidine incorporation studies have shown anti-proliferative activities in the crude ethyl acetate fractions (Figure 1B) and also purified compound (Figure 2B). Flow cytometry analysis of the appearance of distinct sub-G1 peak was seen in the cells treated either with the crude ethyl acetate extract or with the pure compound as early as 48 h and 72 h, more than 70% of cells undergo apoptosis (Figure 1C and 2C). Since apoptotic programme can be manipulated to produce massive changes in cell death, the genes and proteins controlling apoptosis are potential targets. In instances where apoptosis is disabled by proto-oncogenes, agents that disturb their anti-apoptotic function can produce remarkable increases in cell death. The role of c-*myc* in apoptosis is well known (Thompson, 1995). Replicating cells are known to maintain high level of c-*myc* expression correlating with growth and onset of apoptosis (Evan *et al*, 1992). Our results indicate that the both crude ethyl acetate extract and pure compound were capable of increasing the levels of expression c-*myc* (Figure 4), suggesting a possibility of the induction of apoptosis. We have also confirmed the induction of apoptosis in HEp2 cells after treatment with crude ethyl acetate extract and pure compound by Propidium iodide and annexinV staining at 72 h (data not shown). It has been shown earlier that c-*myc* enhances apoptosis in low concentrations of survival factors or oxygen and following treatment with diverse cytotoxic agents (Evan *et al*, 1992; Graeber *et al*, 1996). Although the induction of apoptosis was studied in detail on HEp2 cells, our results with other cell lines tested suggest that both crude ethyl acetate fraction and the pure compound induced cell death as early as 24 h after treatment (Figure 3), suggesting that inducing apoptotic cascade may be a useful target in cancer cells for effective therapeutic measures.

Preliminary results on tubulin during mitotic spindle formation suggest that the pure compound, 7′-hydroxy-3′,4′,5,9,9′-pentamethoxy-3,4-methylene dioxy lignan promotes the assembly of tubulin (Figure 6) after 24 h of treatment of HEp2 cells. Although it has long been argued that drugs that affect microtubule assembly will be effective against tumours through the inhibition of mitosis, all evidence suggest that 7′-hydroxy-3′,4′,5,9,9′-pentamethoxy-3,4-methylene dioxy lignan brings about anti-tumour activity through initiation of apoptosis in cycling cells. Similar effects are observed in previously used agents like taxol, which promote tubulin assembly and inhibit microtubule depolymerisation (Keqiang *et al*, 1998).

It is clear from our results that the above compound induces apoptosis by *bcl2* suppression and activation of caspases (Figure 7). Earlier studies have suggested that suppression of bcl2 activity in cells and activation of caspases 3 and 8 is strictly associated with apoptosis (Kang Boem Kown *et al*, 2001) The inhibition of telomerase by the pure compound may also drive the cells to the apoptotic cascade. However, whether this is due to a direct effect on telomerase will need to be confirmed further, but the data on other targets of apoptosis may indicate that there could be a possible correlation (Figure 5). In an earlier study c-*myc* over expression upregulates telomerase activity in mouse embryonic fibroblasts and myeloid cells (Rachid *et al*, 2001). It appears that the whole process of cell death induction by cytotoxic agents will require the assessment of several closely associated targets in the death cascade. Thus, the measure of telomerase activity alone might not be informative, and has to be studied along with other targets of cell death.

In conclusion, our studies highlight the ability of integrating ethno-botanical leads, chemical isolation and cell signalling cascade targets to screen and isolate molecules with potent anti-cancer activities. This study also indicates that 7′-hydroxy-3′,4′,5,9,9′-pentamethoxy-3,4-methylene dioxy lignan compound induces apoptosis in a wide spectrum of cancer cell line, blocks known anti-apoptotic and stimulates proapoptotic cascades. The possibility of this compound as an effective pharmaceutical product is worth pursuing.
